# Retinol Binding Protein 7 Promotes Adipogenesis *in vitro* and Regulates Expression of Genes Involved in Retinol Metabolism

**DOI:** 10.3389/fcell.2022.876031

**Published:** 2022-04-14

**Authors:** Dong-Hwan Kim, Jinsoo Ahn, Yeunsu Suh, Ouliana Ziouzenkova, Jeong-Woong Lee, Kichoon Lee

**Affiliations:** ^1^ Biotherapeutics Translational Research Center, Korea Research Institute of Bioscience and Biotechnology, Daejeon, South Korea; ^2^ Department of Functional Genomics, University of Science and Technology, Daejeon, South Korea; ^3^ Department of Animal Sciences, The Ohio State University, Columbus, OH, United States; ^4^ Department of Human Sciences, The Ohio State University, Columbus, OH, United States; ^5^ The Ohio State University Interdisciplinary Human Nutrition Program, The Ohio State University, Columbus, OH, United States

**Keywords:** Vitamin A, binding proteins, adipogenesis, retinoid metabolism, lipid metabolism

## Abstract

Retinol is an essential nutrient in animals. Its metabolites, specifically retinoic acid (RA), are crucial for cell differentiation, including adipogenesis. Retinol binding protein 7 (Rbp7) is under the control of PPARγ, the master regulator of adipogenesis. However, the role of RBP7 in adipogenesis is unclear. Our study showed that Rbp7 was abundantly expressed in white and brown mouse adipose tissues and had a higher expression in adipocytes than in stromal vascular fraction. Rbp7 overexpression promoted 3T3-L1 preadipocyte differentiation with increased triglyceride accumulation and up-regulation of Pparγ, Fabp4, C/ebpα, and AdipoQ. Rbp7 deficient adipocytes had opposite effects of the overexpression, which were rescued by RA supplementation. Indirect assessment of relative nuclear RA levels using RAR response element (RARE)-Luc reporter assay demonstrated that *Rbp7* overexpression significantly increased RARE-Luc reporter activity. Rbp7 overexpression significantly increased expression of Raldh1, responsible for RA production, and up-regulation of Lrat and Cyp26a1, involved in retinol storage and RA catabolism, respectively, in 3T3-L1 adipocytes. Rbp7 deficient adipocytes had opposite effects of the overexpression of those genes involved in retinol metabolism. These data suggest that RBP7 increases transcriptional activity of RARE that may induce negative feedback responses via regulation of the gene expression for retinol homeostasis. Our data indicate critical RBP7 functions in adipocytes: regulation of transcriptional activity of RARE and adipocytes differentiation, potentially providing a new target for obesity therapy.

## Introduction

Vitamin A (also known as retinol) is an essential nutrient in animals and has functions in vital physiological actions (e.g. tissue morphogenesis, vision, immune function, etc.) (Ross et al., 2000). Retinol and its metabolites, also termed as retinoids, function as key regulatory molecules that control embryogenesis and differentiation of many types of cells in adults, including adipocytes (Reichert et al., 2011; Kam et al., 2012). Retinol can be esterified by the action of the lecithin:retinol acyltrasferase (LRAT) enzyme and stored in adipose tissues (Wongsiriroj et al., 2014). The reversible oxidation of retinol by alcohol dehydrogenases and short-chain dehydrogenase-reductases yield retinaldehyde (RAL) (D’Aniello and Waxman, 2015). In a second irreversible reaction, RAL is oxidized by retinal dehydrogenases (RALDH1-3, alias: Aldh1A1-3). After all-*trans* retinoic acid (atRA) is synthesized in the cell, it is transported into the nucleus by cellular retinoic acid binding protein II (Sessler and Noy, 2005). In the nucleus, all isomers of retinoic acid (RA) are ligands for retinoic acid receptors (RARs) and the 9-*cis* RA isomer is a ligand for retinoid X receptors (RXRs). Activated RAR and RXR form heterodimer, which regulates expression of genes for diverse functions, including differentiation (Lowe et al., 2011). Adipogenesis requires RAR and RXR during the early and late phases of differentiation (Agarwal et al., 2000; Yamauchi et al., 2001). Consonantly, endogenous RA production in adipocytes is supported by an increased expression of enzymes, RALDH1-3, which regulate preadipocyte survival and adipogenesis (Berry et al., 2012). An excess of generated atRA leads to activation of RAR and its target gene cytochrome P450 family 26 (Cyp26) (Zemany et al., 2014). As a negative feedback pathway, CYP26 catalyzes RA oxidation and degradation.

Retinol binding proteins (RBPs) are a family of carrier proteins for retinol in plasma and cytoplasm. RBPs are involved in retinol transport and can regulate metabolism as signaling molecules. After dietary intake of vitamin A, intestinal cells secrete chylomicron containing retinyl esters (RE). Hepatic uptakes of chylomicron remnants are the major source for RE stored in the hepatic stellate cells (Quadro et al., 1999). From the liver, retinol in complex with RBP4 [alias: serum RBP (sRBP)] and transthyretin (Ttr) is transported into the circulation (Hendriks et al., 1987; Liu et al., 1993). Cellular uptake of retinol from this holo-RBP4 complex is mediated by STRA6 (Duester, 2008; Muenzner et al., 2013). Three types of cellular retinol-binding proteins (CRBPs) are responsible for intracellular trafficking of retinol and its metabolites (Vogel et al., 2001; Belliveau et al., 2010). CRBP-I (alias: RBP1) and CRBP-II (alias: RBP2) were well-characterized in previous studies. *Crbp*-I is widely expressed in various tissues including adipose tissue, where it has an important role in adipocyte differentiation (Zizola et al., 2010). *Crbp*-II is highly expressed in the early but not in the late stage of adipocyte differentiation (Frey and Vogel, 2011). CRBP-III (alias: RBP7 or CRBP-IV in humans (Vogel et al., 2001; Folli et al., 2002)) is involved in lipid and whole-body energy metabolism (Zizola et al., 2008). Previous studies showed that *Rbp7* is specifically expressed in adipose tissues in avian, mice, and pigs (Ahn et al., 2015; Ahn et al., 2018; Ahn et al., 2019a). In mice, *Rbp7* is a target gene of peroxisome proliferator-activated receptor-γ (PPARγ) transcription factor, which is a master regulator of lipid metabolism and adipogenesis (Zizola et al., 2008; Bojková et al., 2010; Chen et al., 2015). Although, our recent study showed that *Rbp7* is expressed in adipose specifically and up- or down-regulated by retinol or atRA, respectively, RBP7 expressions at mRNA and protein levels are significantly increased in BAT during cold exposure (Ahn et al., 2018), RBP7 function in adipocytes still remains unclear.

Adipose tissue plays an important role in both lipid and vitamin A metabolism. Approximately 15% of the total retinoids in the body is stored in adipose tissue as RE (Tsutsumi et al., 1992). The functional understanding of vitamin A and its metabolites, retinol, RAL, and atRA, is incomplete and the proposed mechanisms remain controversial. The dietary studies with vitamin A revealed dose- and species-specific effects on obesity. Mice fed with a very high vitamin A diet had a decreased adiposity (Suryawan and Hu, 1997; Dawson et al., 2000; Jeyakumar et al., 2005). As an *in vitro* model, 3T3-L1 cells are widely used for studies on the process of preadipocyte differentiation (Asano et al., 2014; Jang et al., 2015) and the underlying mechanisms of retinoic acid (Abd Eldaim et al., 2017; Stoecker et al., 2017; Ahn et al., 2018). Supplementation of atRA can lead to inhibition or activation of adipogenesis in the preadipocyte 3T3-L1 model depending on times and concentrations of atRA treatment (Safonova et al., 1994; Suryawan and Hu, 1997; Hida et al., 1998; Dave et al., 2012; Kim et al., 2019). Especially, our recent studies using avian species revealed that atRA induced/promoted adipocyte differentiation *in vitro* (Kim et al., 2020; Lee et al., 2021) and increased adipose tissues during the development of chicken embryos (Kim et al., 2021). The understanding of retinoid functions on adipogenesis comes from studies investigating endogenous metabolism of vitamin A. The deletion of RALDH1 enzyme producing RA decreases adipogenesis *in vivo* and *in vitro* (Reichert et al., 2011). All RBP proteins, that are responsible for retinol delivery (Yang et al., 2005) and intracellular transport (Zizola et al., 2008; Zizola et al., 2010), demonstrated their effects on lipid metabolism in knockout models. However, until now, it is unclear whether RBP proteins can influence RA production and functions in the cytosolic or nuclear compartment of adipocytes. In this study, we investigated the role of RBP7 in adipogenesis and its effects on nuclear availability of RA for gene regulation. The cumulative data indicate that RBP7 promotes adipocyte differentiation and increases nuclear RA levels for regulation of genes maintaining RA and retinol homeostasis.

## Materials and Methods

### Data Mining

The Human Protein Atlas (HPA) RNA-Seq data were obtained from the Array Express Archive (www.ebi.ac.uk/arrayexpress/) with the accession number: E-MTAB-1733 (Fagerberg et al., 2014). Muscle tissue was not available in the HPA dataset. Fourteen human tissues were used in this study. The microarray-based gene expression data were obtained from the GEO DataSet (GDS) of the Gene Expression Omnibus (GEO) repository in the National Center for Biotechnology Information (NCBI) archives (www.ncbi.nlm.nih.gov/geo). For analyzing a tissue distribution pattern of gene expression in 13 male mouse tissues, GDS3142 was downloaded and sorted as described in our previous reports (Song et al., 2013; Zhang et al., 2014). In addition, GDS6247 was used for analyzing changes in expression levels of Rbp7 in adipose tissues by high-fat diet feeding or ageing in mice.

### Animal Use and Sample Preparation

All animal care and procedures were approved by the Institutional Animal Care and Use Committee (IACUC) at The Ohio State University (Protocol number: 2007A0183, Approval date: 01 October 2007). Mice (C57BL/6J) were raised under ad libitum feeding conditions in a mice housing facility at The Ohio State University and tissues were sampled after euthanizing by carbon dioxide inhalation followed by cervical dislocation.

### Western Blot Analysis

Western blot analysis with tissue protein extracts was performed as described in our previous report (Kim et al., 2019). Protein extracts (30 μg) were loaded onto 15% SDS-PAGE gels and transferred to PVDF membranes (Bio-Rad, Hercules, CA). The membranes were blocked with 10% skim milk (#232100, ThermoFisher Scientific, Schwerte, Germany) for 30 min and then incubated with antibodies against murine RBP7 [(Ahn et al., 2015), 1:2,000, AbClon, Korea], PPARγ (#SC-7273, 1:1,000, Santa Cruz Biotechnology, Santa Cruz, CA), FABP4 (#2120, 1:1,000, Cell signaling Technology, Danvers, MA), LRAT (#PA5-38556, 1:1,000, ThermoFisher Scientific), ALDH1A1 (#NB-100-787, 1:500, Novus Biologicals, Littleton, CO), CYP26A1 (#PA5-24602, 1:1,000, ThermoFisher Scientific), or β-Actin (#4970, 1:10,000, Cell signaling Technology) at 4°C overnight. The next day, after washing with PBS containing 0.3% tween 20, an appropriate secondary antibody (HRP-linked anti-rabbit IgG (HAF008); 1:5,000; R&D Systems Inc., Minneapolis, MN) was applied to the membrane before washing and developing with ECL plus reagents and detected by the LAS-3000 luminescent image analyzer system (Fujifilm, Japan). β-Actin was used as an internal control.

### Lentiviral Infection and Transient Transfection

The Rbp7 viral expression vector was constructed by ligating the full-length cDNA coding sequence (CDS) of the mouse into the modified LentiCRISPRv2GFP (#82416, Addgene, Cambridge, MA). In short, the LentiCRISPRv2GFP vector was cut to remove the CRISPRv2GFP by restriction enzymes, KpnI (#R0142, New England Biolabs, Ipswich, MA) and BsrGI (#R0575, New England Biolabs), and an amplified CMV promoter with the mouse Rbp7 CDS was inserted into the vector. Constructed viral vector, pMD2. G and psPAX2 (#12259 and #12260, Addgene) vectors were co-transfected into Lenti-X 293T cells by Lipofectamine 3,000 (#L3000075, ThermoFisher Scientific), according to the manufacturer’s protocol (Invitrogen, Carlsbad, CA). Viral supernatants were harvested at 48 h after transfection and concentrated by ultra-centrifugation. Generally, to induce overexpression of *Rbp7* (OE), polybrene (1:1,000, #SC-134220, Santa Cruz) were treated for 1 h and then Rbp7 viral supernatants were treated for 8 h on 3T3-L1 cells. To induce *Rbp7* knockdown (siRbp7), 3T3-L1 cells were transfected with siRNA (Bioneer, Korea) using Lipofectamine 3,000 (ThermoFisher Scientific). After 48 h, OE, Mock (control), and siRbp7 groups were induced for differentiation with or without all-*trans* retinoic acid (#R2625, Sigma-Aldrich, St. Louis, MO) and used for analysis.

### Cell Fractionation and Culture, RNA Isolation & Reverse Transcription

For the quantification of gene expression in fat cell fractions, subcutaneous adipose tissue was collected from mice. Tissue was fractionated into stromal-vascular cells (SV) and fat cells (FC) as described previously (Song et al., 2013). At the day of cell confluency, OE or siRbp7 was induced on 3T3-L1 cells for 2 days, and then adipogenic differentiation was induced as following previous procedures (Kim et al., 2019). Cells were cultured in a differentiation cocktail (DMEM containing 10% FBS supplemented with 0.25 μM dexamethasone, 0.5 mM 3-isobutyl-1-methylxanthine, and 10 μg/ml insulin), and then the differentiation medium was changed to DMEM containing 10% FBS with 10 μg/ml insulin. A universal siRNA control was used as a negative control. Total RNA was isolated from murine subcutaneous adipose tissue and 3T3-L1 cells using Trizol (Life Technologies Inc., Grand Island, NY) according to the manufacturer’s instructions. RNA quality and quantity were assessed by gel electrophoresis and NanoDrop measurement (NanoDrop Technologies, Wilmington, DE) and the samples were stored at −80°C until use. Approximately 1 μg of RNA was reverse-transcribed in a 20 μL total reaction to cDNA using Moloney murine leukemia virus (M-MLV) reverse transcriptase (Invitrogen). The thermal cycle of the reverse transcription was 65°C for 5 min, 37°C for 52 min, and 70°C for 15 min.

### Real-Time PCR

Quantitative real-time PCR (qPCR) was performed on an ABI 7300 Real-Time PCR instrument (Applied BioSystems, Foster City, CA) by using AmpliTaq Gold polymerase (Applied BioSystems) with SYBR green detection dye. *Cyclophilin* (*Cyc*) was used as a housekeeping gene. Reactions were performed in duplicates as a total volume of 20 μl for each, and conditions for the qPCR were 95°C for 10 min followed by 40 cycles of 94°C for 15 s, 60°C for 40 s, 72°C for 30 s, and 82°C for 33 s. Relative quantification of gene expression was determined by using the 2^−ΔΔ^C_T_ method (Livak and Schmittgen, 2001). Primer sequences are listed in Supplementary Table S1.

### Oil-Red-O Staining

Lipid accumulation was measured in adipocytes using Oil-Red-O (ORO) staining. Differentiated 3T3-L1 adipocytes were fixed with 10% formalin for 1 h and rinsed with 60% isopropanol. The samples were stained with 0.3% ORO solution for 10 min at RT. After rinsing with distilled water, specimens were dried and photographed. To quantify lipid accumulation, the ORO was extracted with 100% isopropanol and collected, and absorbance values were measured at 490 nm by a spectrophotometer (SpectraMax M3, Molecular Devices, Sunnyvale, California).

### Quantification of Triglycerides

Quantification of triglycerides in fully differentiated 3T3-L1 cells was performed using a triglyceride quantification assay kit (#ab65336, Abcam, Cambridge, MA) according to the manufacturer’s instructions. Briefly, cells were harvested and washed with cold PBS. Cells were lysed with 5% NP-40. Cell lysates were heated in boiled water and cooled down at RT, and then amounts of triglycerides were analyzed at 570 nm by a spectrophotometer (SpectraMax M3).

### Luciferase Assay

3T3-L1 cells were transiently transfected with siRbp7 oligos using the AMAXA basic nucleofector (#VPI-1002, Lonza, Basel, Switzerland) and Nucleofector system (Lonza) with a program T-030 for 3T3-L1 following manufacturer’s instructions. Rbp7 viral supernatants were also added into each of the cells to transduce the gene. After 48 h, RARE-Luc and RARα-LBD, described in our previous study to measure RARE activity (Ziouzenkova et al., 2007), and Renilla luciferase vectors (added 6:6:1 v/v/v) were transfected using Lipofectamine 3,000. Luciferase activity was measured 2 days after transfection of the cells and also measured 4 days after transfection of 3T3-L1 cells, which was on day 2 of differentiation, using the Dual-Luciferase assay system (#E1980, Promega, Madison, WI) by Glomax (Promega).

### Statistical Analysis

All data were expressed as means ± S.E.M. Individual experiments were performed at least three times. The data were analyzed using Graphpad Prism software, version 6.02. For comparison of gene expression between two means, a multiple *t*-test was conducted. Also, multiple means were compared by one-way ANOVA followed by Tukey’s multiple comparisons test. The *p*-value, *p* < 0.05, was considered as a statistically significant difference.

## Results

### Adipose-Specific Expression of RBP7 Gene and Protein

**FIGURE 1:**
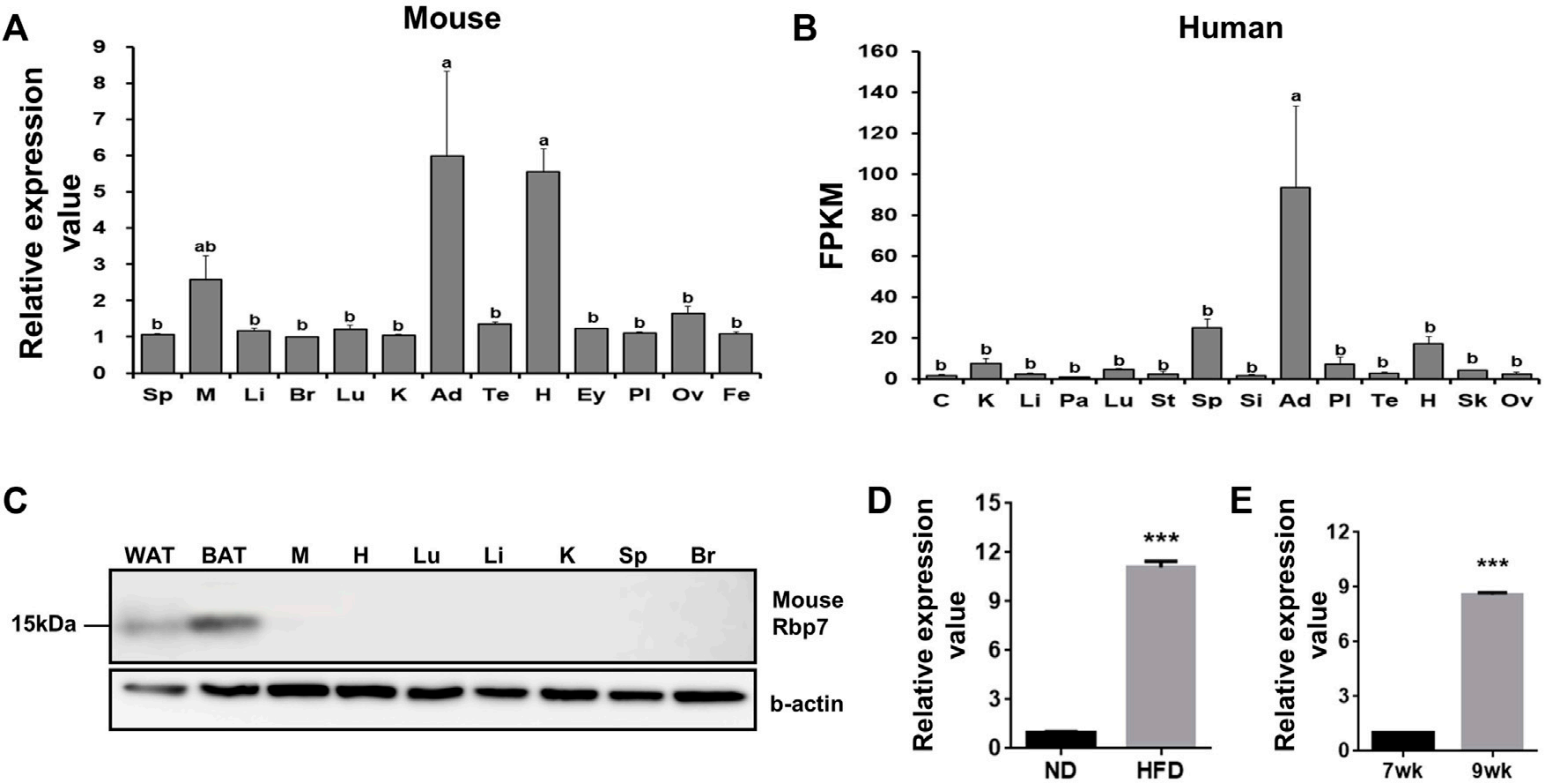
Tissue distribution of Rbp7 expression. **(A,B)** Expression levels of mouse and human *RBP7* in various tissues were presented based on microarray (GEO dataset) and RNA-seq dataset (Human Protein Atlas). **(C)** Western blot analysis was conducted to detect mouse RBP7 protein in various tissues. Sp: spleen, M: muscle, Li: liver, Br: brain, Lu: lung, K: kidney, Ad: adipose tissue, Te: testis, H: heart, Ey: eye, Pl: placenta, Ov: ovary, Fe: fetus, C: colon, Pa: pancreas, St: stomach, Si: small intestine, Sk: skin, WAT: white adipose tissue, BAT: brown adipose tissue. β-actin was used as a loading control for an equal amount of protein extracts. One-way ANOVA followed by Tukey’s multiple comparisons test was used and the bars indicate means ± S.E.M. statistically significant differences are marked by the three letters, a, ab, or b (*p* < 0.05). **(D)** Expression levels of mouse *Rbp7* in epididymal adipose tissues after feeding high-fat diet for 2 weeks. **(E)** Age-related changes in expression levels of *Rbp7* in mouse epididymal adipose tissues. The expression data in D and E were presented based on GEO dataset. *t*-tests were used for statistical analysis by the Graphpad PRISM 6.02 program. The bars indicate means ± S.E.M. ***p < 0.001.

To identify genes specifically expressed in adipose tissue, we analyzed mouse and human microarray data using GEO datasets. Among Rbp family members (*Rbp1*, *Rbp4* and *Rbp7*), *Rbp7* was expressed in adipose and heart tissues over 5-folds in mice compared to the average value of other tissues (Figure 1A). In humans, adipose tissue showed a 90-folds higher expression (Figure 1B). We validated adipose-specific expression of *Rbp7* by measuring protein expression of RBP7 in various mouse tissues by Western blot. An expected size of RBP7 protein at 15 kDa was detected only in white and brown adipose tissues (Figure 1C). Rbp7 mRNA level was increased by 11-folds in epididymal adipose tissues of mice fed high-fat diet for 2 weeks compared to normal diet (Figure 1D). In addition, comparison of levels of RBP7 expression in adipose tissues from 7-week-old and 9-week-old mice showed 8.6-fold increase (Figure 1E).

**FIGURE 2:**
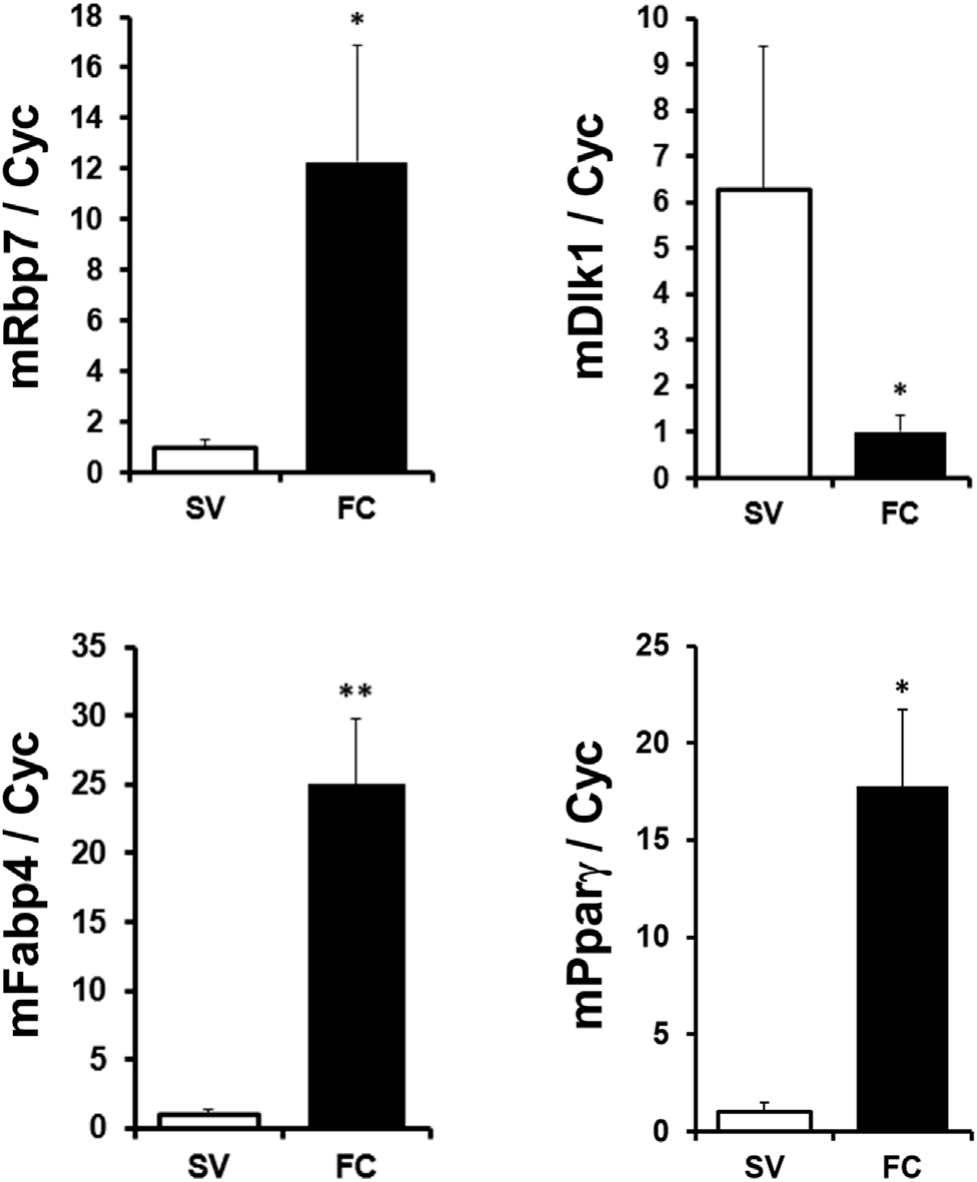
RBP7 expression in stromal vascular cell and fat cell fractions. Subcutaneous adipose tissues were separated to the fractionation of stromal-vascular cell (SV) and fat cell (FC), and analyzed. *Fabp4* and *Pparγ* genes were used as adipocyte markers. *DLK1* was used as a preadipocyte marker. *t*-test was used for statistical analysis by the Graphpad PRISM 6.02 program. The bars indicate means ± S.E.M. *p < 0.05, **p < 0.01.

Next, we compared Rbp7 expression in SV fraction containing mostly preadipocytes and FC fraction containing adipocytes in mouse adipose tissue. Expression levels of adipocyte markers, Fabp4 and Pparγ were 25- and 17-folds higher in the FC fraction, respectively. A preadipocyte marker, Dlk1 (Moon et al., 2002; Lee et al., 2003), was 6-folds lower in FC than in SV (Figure 2), indicating successful fractionation of cells from adipose tissue. Expression levels of Rbp7 in the FC fraction were 12-folds higher than the SV fraction. These expression levels of Rbp7 *in vivo* suggests that Rbp7 is associated with differentiated adipocytes.

### Effect of RBP7 as a Positive Regulator on 3T3-L1 Cell Differentiation

**FIGURE 3:**
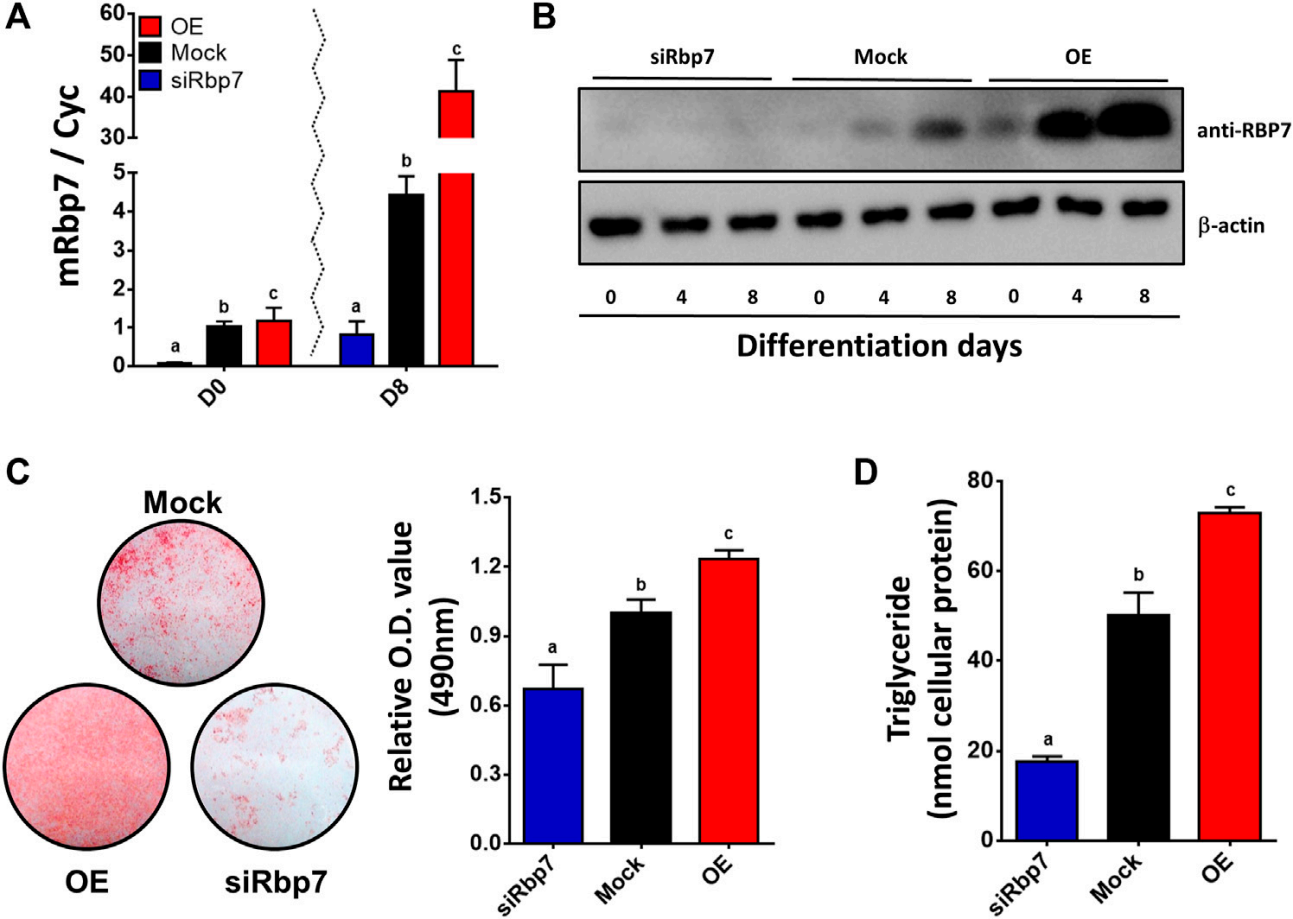
Effect of modulation of Rbp7 expression on adipogenic differentiation of 3T3-L1 cells. **(A)** *Rbp7* expression levels on differentiated 3T3-L1 cells. Red bar: OE group; Black bar: Mock control; Blue bar: siRbp7 group. **(B)** Western blot analysis of mouse Rbp7. Protein extracts (30 μg) were loaded to detect protein expression. β-actin was for an internal control. **(C)** Oil Red O staining. Differentiated 3T3-L1 cells were stained with Oil Red O on day 8 and analyzed the O.D. values by spectrophotometer. **(D)** Quantification of triglyceride. Levels of triglyceride in differentiated 3T3-L1 cells were measured on day 8 by ELISA. One-way ANOVA followed by Tukey’s multiple comparisons test was used for statistical analysis by the Graphpad PRISM 6.02 program. The bars indicate means ± S.E.M. Statistically significant differences are marked by the three letters, a, ab, or b (*p* < 0.05).

To examine the role of *Rbp7* in adipocyte differentiation, 3T3-L1 preadipocytes were infected with the lentiviral supernatants to overexpress mouse *Rbp7* (OE) and transiently transfected with siRbp7 to achieve *Rbp7* knock-down (siRbp7). Two days after transfection, adipogenic differentiation was induced. OE or siRbp7 in 3T3-L1 cells was verified on both mRNA and protein levels that were measured by quantitative real-time PCR and Western blot analyses, respectively (Figures 3A,B). In the mock control, *Rbp7* mRNA and protein expressed at very low levels at day 0 and its expression was increased on day 8 of adipocyte differentiation. OE cells showed significantly higher expression levels of *Rbp7* mRNA and protein compared to the Mock, and the siRbp7 resulted in the expression of very low levels of *Rbp7* mRNA and protein during the entire period of differentiation. Lipid accumulation was significantly increased by Rbp7 overexpression compared to the Mock and siRbp7 groups (Figure 3C). In the same manner, triglyceride (TG) contents were significantly higher in the OE compared to the control (74 nM in the OE and 50 nM in control), however, the siRbp7 showed significantly less levels of triglycerides (17 nM in the siRbp7) (Figure 3D). These morphological and biochemical evidences support the role of RBP7 in positive regulation of lipid accumulation and/or differentiation during 3T3-L1 adipogenesis.

**FIGURE 4:**
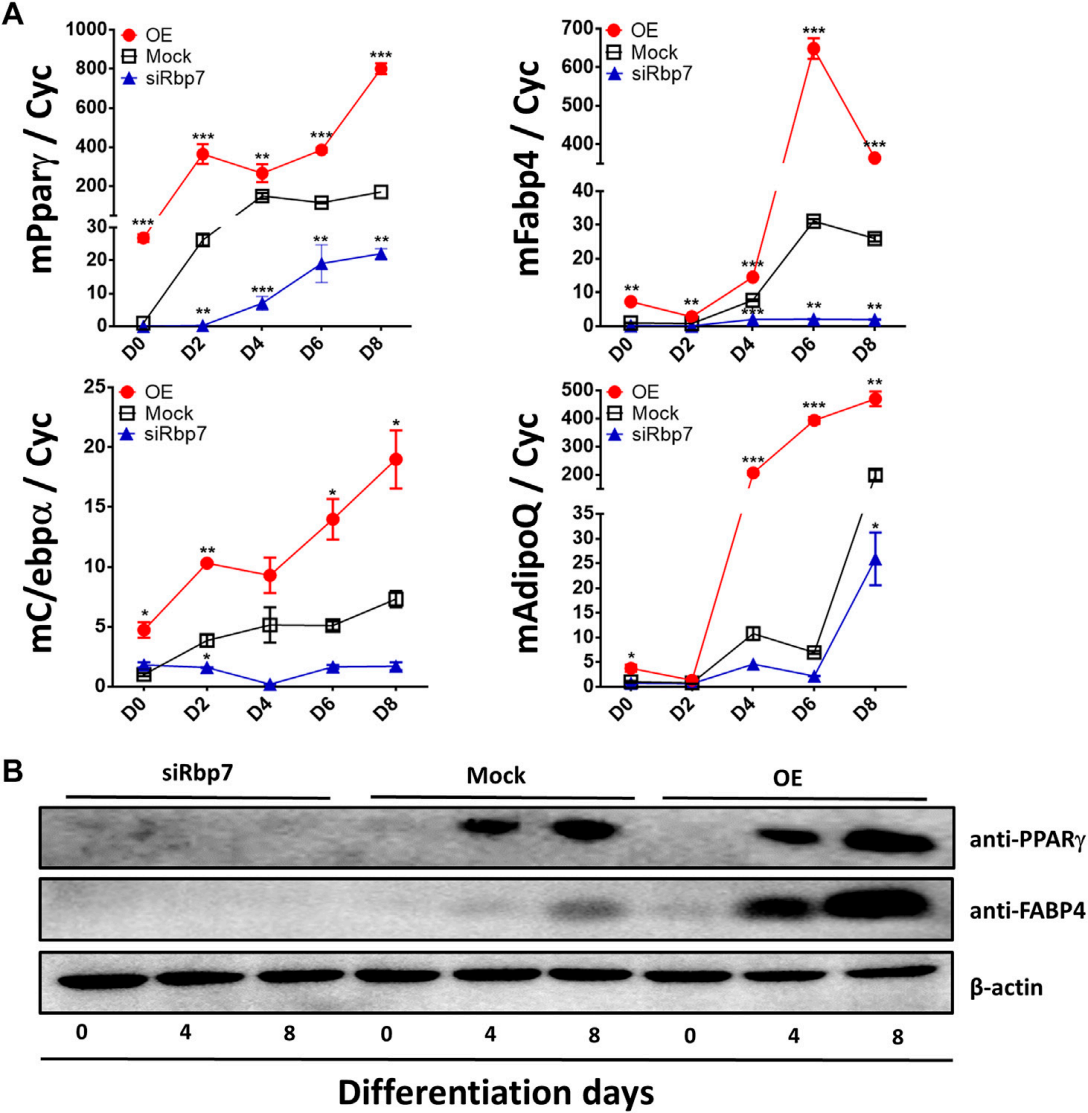
Effects of modulation of Rbp7 expression on expression of adipocyte markers. **(A)** Quantitative analysis of adipocyte markers on differentiated 3T3-L1 cells. *Cyc* was used as housekeeping control. Red circle: OE group; Black rectangular: Mock control; Blue triangle: siRbp7 group. D0-8: preadipocyte differentiation day. Values present means ± SEM. **(B)** Western blot analysis of adipocyte differentiation markers. The data were compared on each time point among different groups (OE vs. Mock vs. siRbp7) to analyze significant difference. One-way ANOVA was used followed by Tukey’s multiple comparisons test. *p < 0.05, **p < 0.01, ***p < 0.001.

Subsequently, expression of adipogenesis-related genes and proteins (Pparγ, Fabp4, C/ebpα or AdipoQ) was further measured by qPCR or Western blot analyses. Expression levels of all these genes were low in preadipocytes and gradually increased by differentiation of 3T3-L1 adipocytes in the control group (Mock, Figure 4A). Adipocytes of the OE group had significantly greater expression levels for all marker genes compared to adipocytes of the Mock (Figure 4A). Agreeably, the cells of the siRbp7 showed decreased expression levels of the adipogenic genes (siRbp7 vs. Mock, Figure 4A). These protein levels and mRNA expression profiles have shown similar patterns in adipocytes with different Rbp7 levels in Figure 3. PPARγ and FABP4 protein levels were gradually increased during differentiation in the Mock and OE; however, expression of these proteins was below detection levels in the siRbp7 (Figure 4B). The highest protein levels of PPARγ and FABP4 were observed by overexpressing Rbp7 (Figure 4B). These data showed that Rbp7 positively regulates murine adipogenesis *in vitro*.

### RBP7 Regulates Transcriptional Activity of Rare and Expression of Genes Involved in Retinoid Metabolism

**FIGURE 5:**
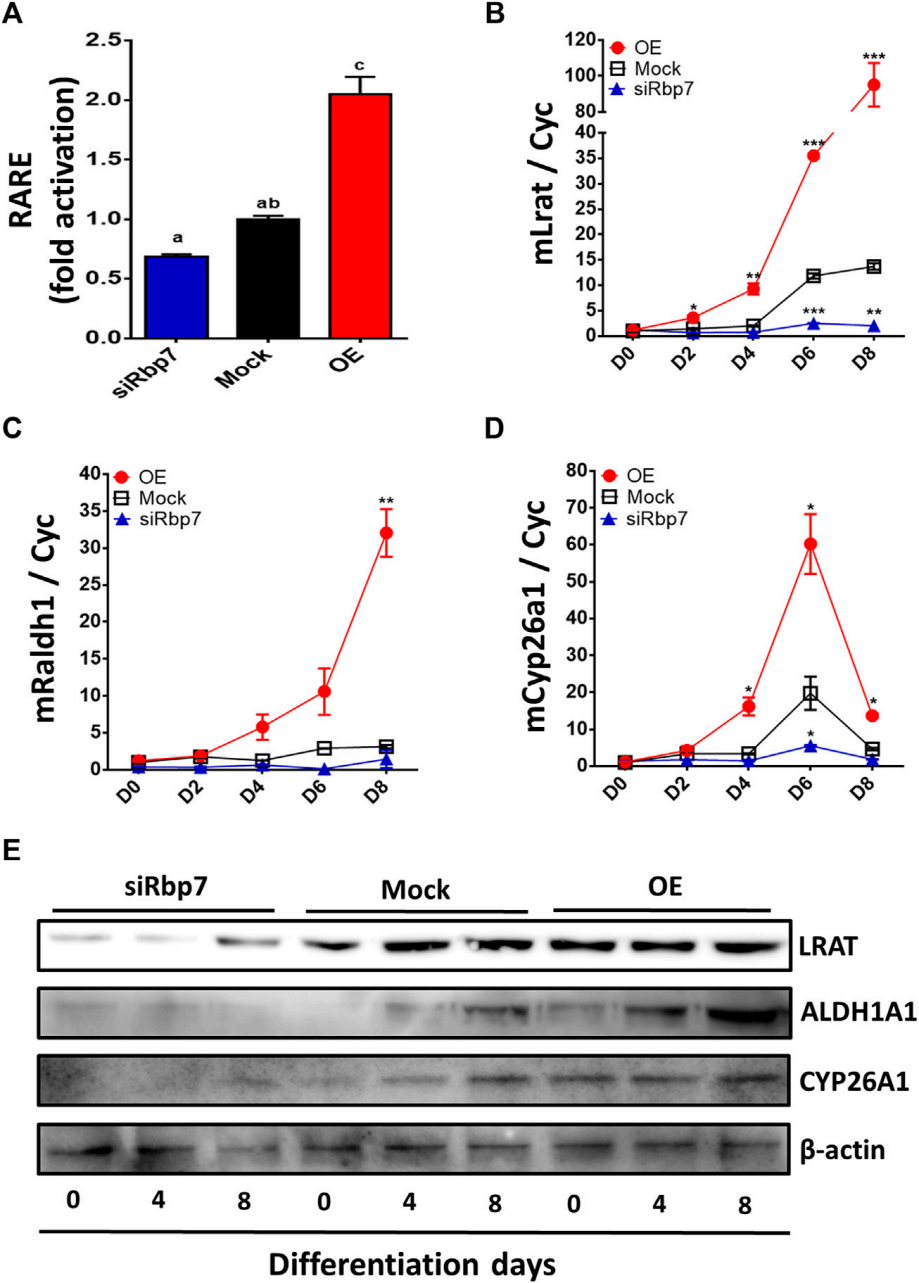
Effects of Rbp7 on nuclear retinoic acid levels and expression of retinoid metabolic genes. **(A)** RARE luciferase activity after 48 h transfection on 3T3-L1 cells. The values were normalized by activity levels of Mock. **(B–E)** Quantitative analysis of diverse markers associated with retinoid metabolism on differentiated 3T3-L1 cells. *Cyc* was used as housekeeping control. Red circle: OE group; Black rectangular: Mock control; Blue triangle: siRbp7 group. D0-8: preadipocyte differentiation day. **(G)** Western blot analysis of retinoid metabolism. Values present means ± SEM. For the statistical analysis, the letters (a, ab, or c) were used to show the significant difference **(A)** and the data were compared on each time point among different groups (OE vs. Mock vs. siRbp7) to analyze significant difference **(B–E)**. All statistical analysis were compared using one-way ANOVA followed by Tukey’s multiple comparisons test. *p < 0.05, **p < 0.01, ***p < 0.001.

The regulation of genes depends on binding of all isomers of RA to RAR/RXR complexes in the nucleus (Germain et al., 2006). Activated RAR/RXR complex binds to the canonic RAR response element (RARE) while inducing gene expression. To measure RA concentrations that support RAR/RXR activation, we employed classic RARE luciferase reporter assay (Hoffman et al., 2006; Zizola et al., 2010). The RARE-Luc reporter was transiently transfected to 3T3-L1 cells expressing different levels of *Rbp7*. RARE was activated in the OE but tended to be suppressed in the siRbp7 compared to the Mock (Figure 5A). These observations suggest that Rbp7 might regulate the transcriptional activity of RARE, participating in the regulation of RAR target genes.

**FIGURE 6:**
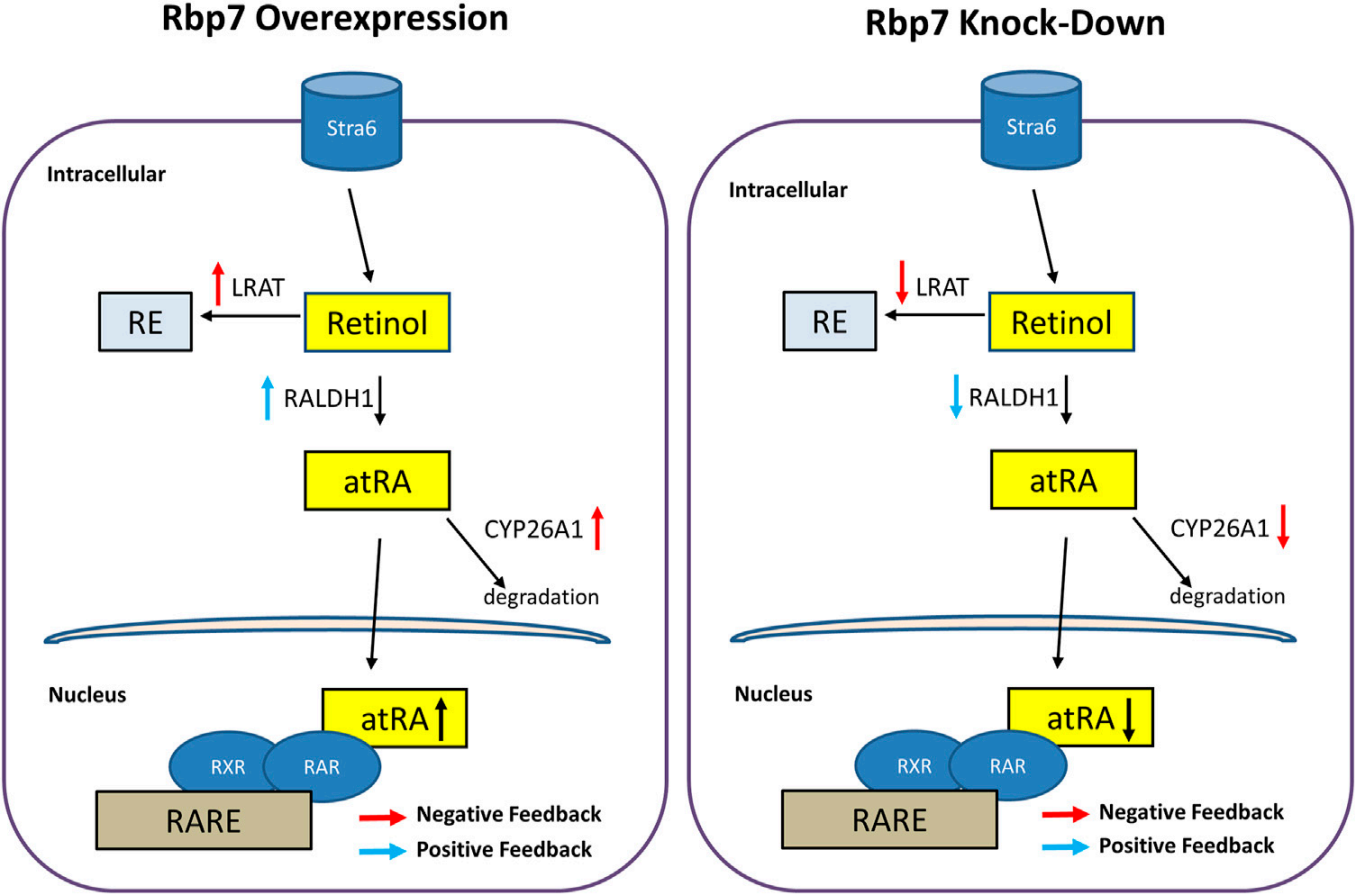
Schematic diagram illustrating hypothetical regulation of nuclear RA activities and expressions of genes/proteins involved in the retinoid metabolism by Rbp7. When Rbp7 is overexpressed, LRAT and CYP26A1 proteins are up-regulated and RALDH1 is down-regulated which might be caused by negative or positive feedback of increased atRA, respectively. In contrast, knock-down of Rbp7 induces down-regulations of LRAT and CYP26A1 and up-regulation of RALDH1 via the feedback mechanisms.

To further investigate the effect of RBP7 on expression of genes involved in retinol metabolism, we analyzed expression levels of genes regulating retinol storage (Lrat), RA synthesis (Raldh1), and RA degradation (Cyp26a1). Expression level of Lrat was gradually increased during 3T3-L1 adipocyte differentiation in the Mock and further up-regulated in the OE (90-folds), but down-regulated in the siRbp7 (2-folds) compared to the Mock (14-folds) at day 8 (Figure 5B). The expression level of Raldh1 was 30-times higher in the OE compared to the Mock at day 8 of the adipocyte differentiation (Figure 5C). Cyp26a1 gene was up-regulated by Rbp7 (Figure 5D). In agreement with the above mRNA data, the protein expression levels for LRAT, ALDH1A1, and CYP26A1 were similar to the mRNA expression levels (Figure 5E). These changes in expression of the genes are probably due to the increased demand of RA during adipogenesis *in vitro*, and also potentially activate metabolic feedback for RA homeostasis (Figure 6).

### RA Addition Rescues the Effect of RBP7 Knock-Down in Adipocytes

**FIGURE 7:**
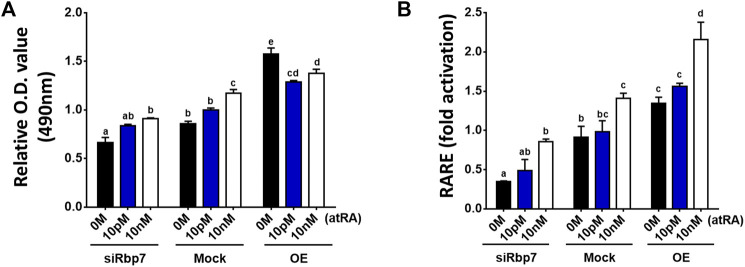
RA addition rescues the effect of Rbp7 knock-down in adipocytes. **(A)** O.D. values of lipid droplets-associated Oil Red O. all-*trans* retinoic acid (atRA) treated siRbp7, Mock, and OE groups were stained with Oil Red O on day 8 of differentiation and analyzed the O.D. values by spectrophotometer. atRA was supplied dose dependently (0 M–10 nM) during the entire period of the preadipocyte differentiation. **(B)** RARE luciferase activity on 3T3-L1 cells. Rbp7 knock-down (siRbp7) and overexpression (OE) was induced by siRbp7 transfection and treatment of Rpb7 viral supernatants 2 days before atRA supplement into adipocyte differentiation medium dose dependently (0 M–10 nM) for 48 h. The value was normalized by activity levels of control (Mock 0 M), which did not induce Rbp7 knock-down/overexpression and cultured without atRA. Significant differences were analyzed by the control (Mock 0 M) in Figures 6A,B. One-way ANOVA followed by Tukey’s multiple comparisons test was used and the bars indicate means ± S.E.M. Statistically significant differences are marked by the letters (*p* < 0.05).

To test the association between RBP7 and transcriptional activity of RARE, adipogenesis was induced on 3T3-L1 cells using the differentiation medium supplemented with all-*trans* RA (atRA, #R2625, Sigma-Aldrich, St. Louis, MO) for the entire period of the preadipocyte differentiation. We used low doses (0–10 nM) of atRA for stimulation to mimic physiologic conditions (Safonova et al., 1994). *Rbp7* deficient cells showed lesser lipid accumulation compared to the control (siRbp7 vs. Mock in 0M atRA), however, these anti-adipogenic effects of siRbp7 were rescued by stimulation with atRA (Figure 7A). In a dose dependent manner, atRA increased lipid droplet accumulations in *Rbp7* deficient 3T3-L1 adipocytes. To the contrary, regardless of RA supplementation, Rbp7 overexpression enhanced lipid accumulation compared to both the control and *Rbp7* knock-down cells. Within the OE groups, lipid accumulation was decreased by supplementation of atRA, suggesting a neutralizing effect of RA oversupply on the accretion of lipid. Lipid accumulation tended to be similar between 0 M of atRA in the Mock and 10 nM of atRA in the siRbp7 (Figure 7A).

The changes in lipid accumulation which corresponded to the alterations in endogenous RA levels (Figure 7A) were further assessed by RARE-Luc activation because it is known that RARE activation is induced by atRA, if they are present in the nucleus (Schräder et al., 1993). RARE-activity was decreased in Rbp7-deficient adipocytes compared to the Mock (siRbp7 vs. Mock in 0M atRA, Figure 7B). Stimulation with atRA increased RARE-activity in a dose-dependent manner in all groups, (siRbp7, Mock, and OE). The siRbp7 cells tended to have a similar level of RARE compared to 0 M of atRA in the Mock by stimulation with 10 nM of atRA (Figure 7B), that was sufficient concentration of atRA to induce the same levels of lipid accumulation (10 nM of RA in siRbp7 vs. 0 M of atRA in Mock, Figure 7A). These results suggest that the effect of Rbp7 on lipid accumulation might depend on the RA production in adipocytes.

## Discussion

Vitamin A metabolites regulate a plethora of functions in various tissues (Owusu and Ross, 2016). Consequently, both deficiency and excess in vitamin A as well as malfunction in the convention of vitamin A to atRA can lead to death. In adipose tissue, deregulation of vitamin A metabolism alters energy homeostasis, cell differentiation, and innervation (Yasmeen et al., 2012; Shen et al., 2018). Retinol is the principal substrate for enzymatic reactions leading to production of RAL and RA as well as RE for storage in lipid droplets. The retinol delivery by RBPs/CRBPs expects to be rate limiting for these enzymatic reactions. However, how RBPs/CRBPs are linked to production of vitamin A derivatives was unclear. Here we provide the evidence of the RBP7-dependent pathway that facilitates RA production for the regulation of adipogenesis. Overproduction of RA was counterbalanced via the RAR-dependent feedback mechanism. Activation of RAR by RA leads to the *Cyp26a1* expression, which is known to induce RA degradation. Previously, coordinated mechanisms for retinol uptake and subsequent esterification have been explained by functional coupling between cytosolic RBP4/STRA6 and LRAT (Montedonico et al., 2008; Amengual et al., 2012). In the adipocytes, RBP7 appears to initiate functional coupling of proteins that are involved in the generation of RA ligands for transcription. In this pathway, unused or excessive intracellular retinol might be esterified by LRAT.

The function of intracellular RBPs in binding and transporting of retinol appears to be redundant (Lee et al., 2016; Abd Eldaim et al., 2017; Napoli, 2017). However, all RBPs have specific expression patterns in different tissues, which are varied among species. It is possible that RBPs can couple different pathways depending on retinol transport and, thereby, support specific metabolic needs and gene regulation in various tissues (Rendenbach et al., 2013; Kanoriya et al., 2016). In mice and humans, our analysis using GEO data sets revealed that RBP1/CRBP-I is broadly expressed, but the ovary, eye, and placenta have the highest expression levels (Supplementary Figure S1). However, RBP7 is expressed in even more specific tissues. We have previously demonstrated *Rbp7* is the adipose-specific expressing gene in the avian species (Ahn et al., 2015). In this study, analysis of GEO datasets and protein expression data revealed that RBP7 is abundantly expressed in white and brown adipose tissues in mice. Although a considerable amount of mouse *Rbp7* mRNA expression was detected in the heart and muscle, RBP7 protein was not detectable in these tissues. In our experiments, we avoided pericardial and intramuscular fat contamination from muscle tissues collected for Western blot analysis. Possible reasons for the discrepancy between our protein data and mRNA expression data from the GEO datasets are the presence of fat in these tissues. Another reason can be low translational efficiencies or high degradation rates of RBP7 protein in these tissues. In humans, *Rbp7* is also predominantly expressed in adipose tissue (Figure 1B), which is further supported by our recent report (Ahn et al., 2019b). The conservation of adipose-specific expression of *Rbp7* in avian and mammals suggests the evolutionary importance of RBP7 functions in adipose tissue.

In our study, lines of evidence revealed that RBP7 is required for adipogenesis. We found higher expression levels of *Rbp7* in the fractionated adipocytes than in the stromal vascular cells in adipose tissue. The loss- and gain-of-function experiments in 3T3-L1 adipocytes support the causative effects of *Rbp7* on adipogenesis and retinol metabolism. *Rbp7* overexpression in 3T3-L1 cells increased formation of lipid droplets and TG accumulation with increased expression of key adipogenic proteins *Pparγ*, *Fabp4*, *C/ebpα,* and *AdipoQ* (Siersbaek et al., 2010; Rong et al., 2011; Chen et al., 2015; Hu et al., 2017). These traits were lost in *Rbp7* knock-down adipocytes suggesting that RBP7 function is critical for adipogenesis *in vitro*.

In our studies, regulation of adipogenesis by RBP7 was coupled to the function of RBP7 in retinoid metabolism, although specific details of this regulation need to be clarified in the future. The unexpected aspect of our study was the discovered link between *Rbp7* expression and transcriptional activity of RARE. *Rbp7* overexpression-driven adipogenesis in 3T3-L1 cells was accompanied by increased RARE activation (Figure 5A), suggesting increased availability of RA isomers or RAR/RXR activation. The causative relation between RA production and adipogenesis was observed in *Rbp7* knock-down adipocytes. The down-regulated *Rbp7* expression prevented lipid accumulation in adipocytes; however, the lipid accumulation was restored by supplementation of atRA after concentration of RA reached endogenous levels. This rescue effect was dependent on the RA dose (Figure 7). Our data suggest that the moderately increased transcriptional activity of RARE might represent a potential role of regulating adipogenic responses that is contributed by RBP7.

RBP7 effect on RA generation is likely indirect. *Rbp7* overexpression increased expression levels of *Raldh1* (alias: *Aldh1a1*). RALDH1 is the major enzyme producing RA for transcriptional activation of RAR in the adipocyte’s nuclei (Reichert et al., 2011). Genetic deficiency in *Raldh1* or pharmacological inhibition of the RALDH1 inhibits adipogenesis *in vivo* and *in vitro*; however, this inhibition could be reversed with physiologic levels of RA (Ziouzenkova et al., 2007). The RA-related pathways in adipose tissue may influence the whole body metabolism as shown in both *Raldh1* ^−/−^ mice and wild type obese mice with implanted *Raldh1*-deficient adipocytes that resisted diet-induced obesity (Reichert et al., 2011; Haenisch et al., 2018). The mechanism linking *Rbp7* to *Raldh1* expression is unknown and its investigation was beyond the scope of the current investigation. A plausible scenario includes binding of retinol by Rbp7 and retinol-dependent stimulation of JNK/MAPK pathway (Du et al., 2017), which increases *Raldh1* expression (Wang et al., 2011). With the point of view in general positive metabolic feedback loop, it is possible that RBP7 can increase available cellular retinol and retinal (Napoli, 2017; Blaner, 2019) a substrate of Raldh1, which might promote production of atRA. Regardless of this speculation, our data show that RBP7 functions upstream of *Raldh1* and adipogenesis. Taken together, nutritional (retinol) and genetic (*Rbp7*) factors might lead to an increase in transcriptional activity of RARE for differentiation of adipocytes.

This study clearly showed increased transcriptional activity of RARE by *Rbp7* overexpression through increased RARE levels (Figure 5A). In addition to promotion of adipogenesis by Rbp7 through the increased transcriptional activity of RARE, the activity might trigger feedback signal to maintain retinol homeostasis in adipocytes (Figure 6). In the current study, increased *Lrat* by *Rbp7* might be mediated through increased transcriptional activity of RARE, as supported by the finding that induction of *Lrat* expression and consequently, an increased storage form of retinol by RA (Ross et al., 2011; Napoli, 2012). In addition, the increased transcriptional activity of RARE by *Rbp7* might activate feedback inhibition by increasing expression of *Cyp26a1* for degradation of excess RA in adipocytes (Ross et al., 2011). These feedback regulations in gene expression by *Rbp7*, leading to RE synthesis for storage and degradation of RA, further support metabolic activities to maintain retinol homeostasis against the increased transcriptional activity of RARE by Rbp7. However, it is possible that the feedback regulations are likely independent of potential pro-adipogenic function of *Rbp7*. At present, it is unclear if these changes in gene/protein expression also correspond to altered enzymatic conversion of retinol such as RE or RAL. Indirectly, this hypothetical mechanism might be supported by evidence of simultaneous occurrence of RA for transcriptional activation and formation of lipid droplets storing lipids and RE (retinol/LRAT axes). RBP7 can shift retinol and lipid homeostasis to produce fat droplet phenotypes in adipocytes. The specific expression pattern of *Rbp7* in adipose tissues combined with its effects on lipid accumulation suggests that RBP7 is a promising candidate for development of anti-obesity therapies.

## Data Availability

The original contributions presented in the study are included in the article/Supplementary Material, further inquiries can be directed to the corresponding authors.
