# Supplementary material for: Retinol Binding Protein 7 Promotes Adipogenesis in vitro and Regulates Expression of Genes Involved in Retinol Metabolism
Source: Front Cell Dev Biol. 2022 Apr 14;10:876031. doi: 10.3389/fcell.2022.876031 (PMC9047791; doi:10.3389/fcell.2022.876031)
Supplement: Supplementary file 1 [file Table1.DOCX]

Table S1. Primers, Assay-on-demand, and siRNA oligonucleotides used in this study

|  | Gene | Forward (5’ to 3’) | Reverse (5’ to 3’) |
| --- | --- | --- | --- |
| qPCR | mRbp7 | GACTCCTTCACCATCCAGACG | TCTTCTCTCCTCTCTGTACGCA |
|  | mDlk1 | AAGGAACCATGGCAGTGCATC | CAGGTCCACGCAAGTTCCAT |
|  | mPPARg | GAAACTCTGGGAGATTCTCCT | CAGAGCTGATTCCGAAGTTGG |
|  | mFABP4 | GATGCCTTTGTGGGAACCTG | TCCTGTCGTCTGCGGTGATT |
|  | mC/ebpα | TGGACAAGAACAGCAACGAGT | GTCAACTCCAGCACCTTCTGT |
|  | mAdipoQ | AATGGCACACCAGGCCGTGAT | TCTCCAGGCTCTCCTTTCCTG |
|  | mLrat | GAGGAGGCACAGGGAAGAAC | ATTAGATGGGCGACACGGTTT |
|  | mRaldh1 | TCCACCCGGAGTCGTCAATAT | CAGCGTAGTCCAAGTCAGCAT |
|  | mCyp26A1 | CCGCTTTATAGTGCCTCATCCA | AGTGGGGCTTGTCTTCATTGT |
|  | mCyclophilin | GGTGGAGAGAGCACCAAGACAGA | GCCGGAGTCGACAATGATG |
|  |  |  |  |
| siRNA | Negative control | UUCUCCGAACGUGUCACGUTT | ACGUGACACGUUCGGAGAATT |
|  | siRbp7 | UCAUUGGAAAGAUGUCACA | UGUGACAUCUUUCCAAUGA |
