# Supplementary material for: Retinol Binding Protein 7 Promotes Adipogenesis in vitro and Regulates Expression of Genes Involved in Retinol Metabolism
Source: Front Cell Dev Biol. 2022 Apr 14;10:876031. doi: 10.3389/fcell.2022.876031 (PMC9047791; doi:10.3389/fcell.2022.876031)
Supplement: Supplementary file 2 [file Presentation1.PPTX]

## Slide 1
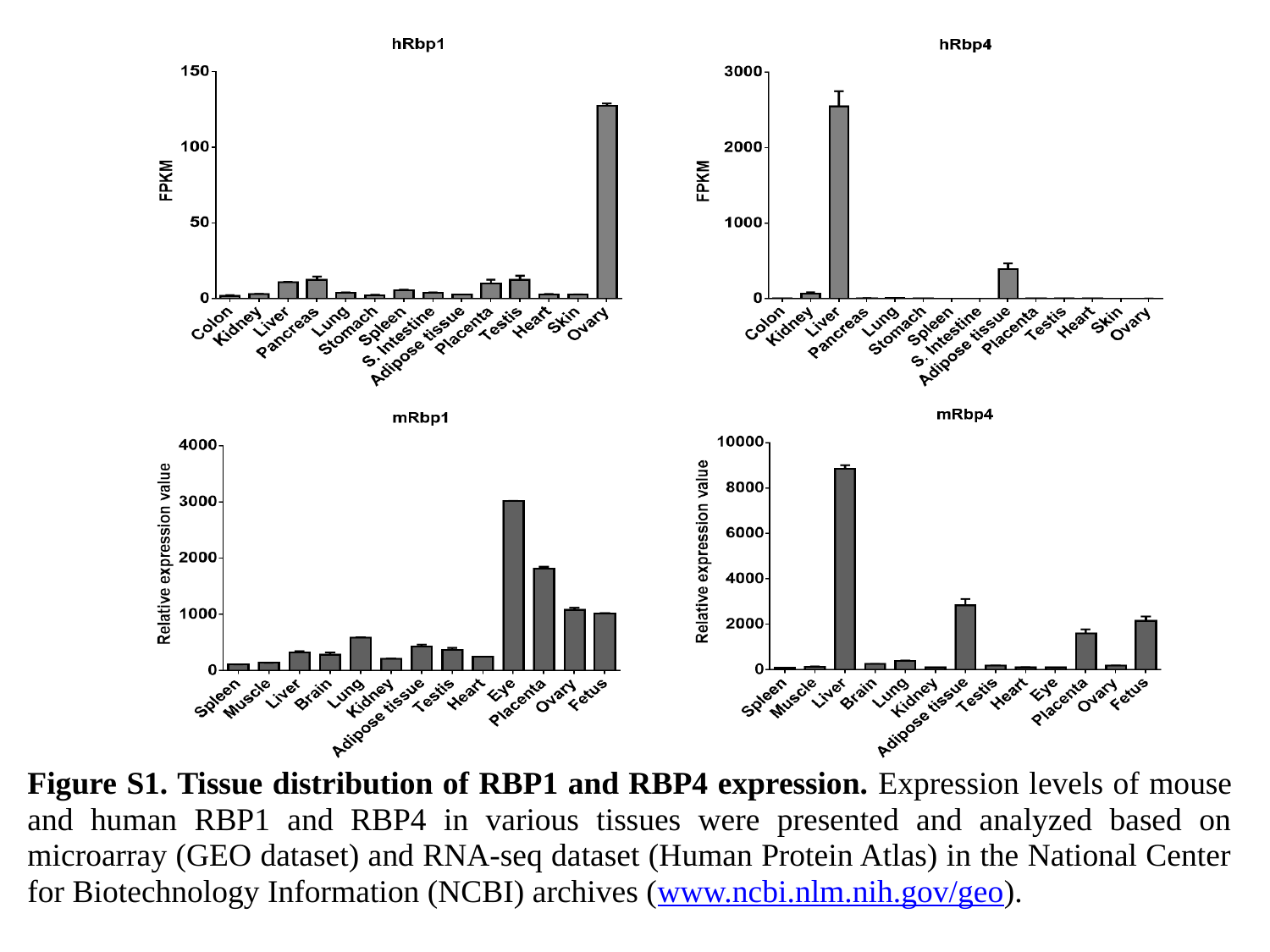

Figure S1. Tissue distribution of RBP1 and RBP4 expression. Expression levels of mouse and human RBP1 and RBP4 in various tissues were presented and analyzed based on microarray (GEO dataset) and RNA-seq dataset (Human Protein Atlas) in the National Center for Biotechnology Information (NCBI) archives (www.ncbi.nlm.nih.gov/geo).
